# Supplementary material for: Meta-Analysis and Gene Set Enrichment Relative to ER Status Reveal Elevated Activity of MYC and E2F in the “Basal” Breast Cancer Subgroup
Source: PLoS One. 2009 Mar 9;4(3):e4710. doi: 10.1371/journal.pone.0004710 (PMC2650420; doi:10.1371/journal.pone.0004710)
Supplement: Dataset S1 — GSEA results from first screen. The file contains a zipped archive of the HTML results of the second GSEA screen. Reports for enriched or depleted datasets may be accessed from the “index.html” link within the my_analysis.GseaPreranked.1219975950625.rpt.zip ZIP archive. Enrichment plots are available for each of the 50 most enriched and depleted gene sets. (13.32 MB ZIP) [file pone.0004710.s001.zip › my_analysis.GseaPreranked.1219975950625.rpt.zip!//LE_MYELIN_UP.html]

Details for gene set LE\_MYELIN\_UP[GSEA]

|  || Dataset | zSco.ERneg.to.ERpos\_collapsed |
| Phenotype | NoPhenotypeAvailable |
| Upregulated in class | na\_pos |
| GeneSet | LE\_MYELIN\_UP |
| Enrichment Score (ES) | 0.6109007 |
| Normalized Enrichment Score (NES) | 2.4489498 |
| Nominal p-value | 0.0 |
| FDR q-value | 0.0 |
| FWER p-Value | 0.0 |
Table: GSEA Results Summary

  

Fig 1: Enrichment plot: LE\_MYELIN\_UP      
 Profile of the Running ES Score & Positions of GeneSet Members on the Rank Ordered List

  

| PROBE | GENE SYMBOL | GENE\_TITLE | RANK IN GENE LIST | RANK METRIC SCORE | RUNNING ES | CORE ENRICHMENT || 1 | CDC20 |  |  | 9 | 7.151 | 0.0289 | Yes |
| 2 | MCM5 |  |  | 31 | 6.533 | 0.0543 | Yes |
| 3 | HDAC2 |  |  | 32 | 6.511 | 0.0812 | Yes |
| 4 | BUB1 |  |  | 41 | 6.445 | 0.1072 | Yes |
| 5 | CCNA2 |  |  | 52 | 6.297 | 0.1325 | Yes |
| 6 | MCAM |  |  | 171 | 5.471 | 0.1462 | Yes |
| 7 | COTL1 |  |  | 178 | 5.444 | 0.1682 | Yes |
| 8 | MCM4 |  |  | 180 | 5.436 | 0.1906 | Yes |
| 9 | CDCA8 |  |  | 194 | 5.328 | 0.2116 | Yes |
| 10 | BIRC5 |  |  | 229 | 5.191 | 0.2305 | Yes |
| 11 | CKS2 |  |  | 239 | 5.145 | 0.2511 | Yes |
| 12 | TUBB6 |  |  | 264 | 5.044 | 0.2701 | Yes |
| 13 | MCM7 |  |  | 289 | 4.970 | 0.2888 | Yes |
| 14 | CORO1C |  |  | 299 | 4.934 | 0.3085 | Yes |
| 15 | EZH2 |  |  | 312 | 4.888 | 0.3278 | Yes |
| 16 | KIF20A |  |  | 318 | 4.874 | 0.3476 | Yes |
| 17 | CKS1B |  |  | 416 | 4.638 | 0.3594 | Yes |
| 18 | CCNB2 |  |  | 431 | 4.589 | 0.3773 | Yes |
| 19 | NT5DC2 |  |  | 453 | 4.499 | 0.3943 | Yes |
| 20 | AURKA |  |  | 460 | 4.483 | 0.4124 | Yes |
| 21 | MAD2L1 |  |  | 477 | 4.446 | 0.4296 | Yes |
| 22 | TNFRSF21 |  |  | 504 | 4.342 | 0.4455 | Yes |
| 23 | RAD51 |  |  | 508 | 4.332 | 0.4632 | Yes |
| 24 | PPP1R14B |  |  | 522 | 4.303 | 0.4800 | Yes |
| 25 | PLK4 |  |  | 524 | 4.292 | 0.4977 | Yes |
| 26 | SMC2 |  |  | 651 | 4.028 | 0.5048 | Yes |
| 27 | CSRP2 |  |  | 706 | 3.916 | 0.5169 | Yes |
| 28 | KIF11 |  |  | 713 | 3.909 | 0.5326 | Yes |
| 29 | PLEKHO1 |  |  | 738 | 3.863 | 0.5467 | Yes |
| 30 | CDC7 |  |  | 769 | 3.807 | 0.5602 | Yes |
| 31 | NUSAP1 |  |  | 836 | 3.732 | 0.5706 | Yes |
| 32 | H2AFX |  |  | 930 | 3.591 | 0.5784 | Yes |
| 33 | PLK1 |  |  | 988 | 3.499 | 0.5886 | Yes |
| 34 | KIF23 |  |  | 1030 | 3.442 | 0.5997 | Yes |
| 35 | LSM2 |  |  | 1098 | 3.364 | 0.6086 | Yes |
| 36 | DNAJC9 |  |  | 1243 | 3.198 | 0.6109 | Yes |
| 37 | CCL2 |  |  | 1664 | 2.791 | 0.5907 | No |
| 38 | SH3GL3 |  |  | 1764 | 2.717 | 0.5945 | No |
| 39 | ECT2 |  |  | 2026 | 2.509 | 0.5851 | No |
| 40 | RRM2 |  |  | 2047 | 2.485 | 0.5939 | No |
| 41 | HMGA2 |  |  | 2058 | 2.478 | 0.6033 | No |
| 42 | H2AFZ |  |  | 2105 | 2.447 | 0.6100 | No |
| 43 | SOX2 |  |  | 2732 | 2.074 | 0.5713 | No |
| 44 | LMNB1 |  |  | 2764 | 2.060 | 0.5774 | No |
| 45 | CXCR4 |  |  | 3062 | 1.902 | 0.5629 | No |
| 46 | ATF3 |  |  | 3455 | 1.727 | 0.5404 | No |
| 47 | CD44 |  |  | 3638 | 1.645 | 0.5334 | No |
| 48 | PDE8A |  |  | 3689 | 1.624 | 0.5364 | No |
| 49 | ETS1 |  |  | 3729 | 1.611 | 0.5401 | No |
| 50 | TOP2A |  |  | 3735 | 1.610 | 0.5464 | No |
| 51 | CDKN1C |  |  | 3739 | 1.609 | 0.5528 | No |
| 52 | TNFAIP8 |  |  | 4451 | 1.334 | 0.5046 | No |
| 53 | EPHA5 |  |  | 4652 | 1.268 | 0.4947 | No |
| 54 | RRM1 |  |  | 4872 | 1.197 | 0.4831 | No |
| 55 | HN1 |  |  | 4953 | 1.171 | 0.4819 | No |
| 56 | CHL1 |  |  | 4958 | 1.169 | 0.4865 | No |
| 57 | TNC |  |  | 5078 | 1.123 | 0.4821 | No |
| 58 | IGSF4 |  |  | 5403 | 1.010 | 0.4618 | No |
| 59 | PSPC1 |  |  | 5515 | 0.969 | 0.4574 | No |
| 60 | POU3F1 |  |  | 5694 | 0.914 | 0.4478 | No |
| 61 | INCENP |  |  | 5907 | 0.845 | 0.4352 | No |
| 62 | ODZ3 |  |  | 6070 | 0.791 | 0.4263 | No |
| 63 | SPRED2 |  |  | 6361 | 0.698 | 0.4072 | No |
| 64 | FOXD3 |  |  | 7596 | 0.270 | 0.3151 | No |
| 65 | EDNRB |  |  | 7927 | 0.136 | 0.2908 | No |
| 66 | COL18A1 |  |  | 8519 | -0.087 | 0.2465 | No |
| 67 | BZW2 |  |  | 8676 | -0.160 | 0.2354 | No |
| 68 | KIF22 |  |  | 8725 | -0.180 | 0.2325 | No |
| 69 | VIL2 |  |  | 8844 | -0.228 | 0.2245 | No |
| 70 | ID2 |  |  | 9049 | -0.335 | 0.2105 | No |
| 71 | RPL13A |  |  | 9091 | -0.358 | 0.2089 | No |
| 72 | MARCKSL1 |  |  | 9116 | -0.368 | 0.2086 | No |
| 73 | NOLA2 |  |  | 9385 | -0.499 | 0.1904 | No |
| 74 | HNRPA2B1 |  |  | 9531 | -0.578 | 0.1819 | No |
| 75 | BASP1 |  |  | 10394 | -1.088 | 0.1212 | No |
| 76 | KLF10 |  |  | 10417 | -1.100 | 0.1241 | No |
| 77 | CUEDC2 |  |  | 10814 | -1.401 | 0.1000 | No |
| 78 | RPA3 |  |  | 11357 | -1.892 | 0.0669 | No |
| 79 | CXCL14 |  |  | 12915 | -4.187 | -0.0334 | No |
| 80 | REEP1 |  |  | 13155 | -5.073 | -0.0305 | No |
| 81 | CCND1 |  |  | 13160 | -5.098 | -0.0098 | No |
| 82 | P2RX4 |  |  | 13183 | -5.269 | 0.0103 | No |
Table: GSEA details [plain text format]

  

Fig 2: LE\_MYELIN\_UP: Random ES distribution      
 Gene set null distribution of ES for **LE\_MYELIN\_UP**
